# Value of p53 sequencing in the prognostication of head and neck cancer: a systematic review and meta-analysis

**DOI:** 10.1038/s41598-022-25291-2

**Published:** 2022-12-01

**Authors:** Shadi Basyuni, Gareth Nugent, Ashley Ferro, Eleanor Barker, Ian Reddin, Oliver Jones, Matt Lechner, Ben O’Leary, Terry Jones, Liam Masterson, Tim Fenton, Andrew Schache

**Affiliations:** 1 Genomics England Head and Neck Cancer Clinical Interpretation Partnership, England, UK; 2 Department of Oral and Maxillo-Facial Surgery, Cambridge University Hospitals, Cambridge, CB2 0QQ UK; 3 Medical Library, School of Clinical Medicine, University of Cambridge, Cambridge, UK; 4 Faculty of Medicine, University of Southampton, Southampton, UK; 5 University of Durham, Durham, UK; 6 Cancer Institute, Faculty of Medical Sciences, University College London, London, UK; 7 The Institute of Cancer Research, London, UK; 8 Liverpool Head & Neck Centre, University of Liverpool, Liverpool, UK; 9 Department of Ear, Nose and Throat Surgery, Cambridge University Hospitals, Cambridge, UK

**Keywords:** Cancer, Head and neck cancer, Surgical oncology, Oncology, Head and neck cancer

## Abstract

This review aimed to examine the relationship between TP53 mutational status, as determined by genomic sequencing, and survival in squamous cell carcinoma of the head and neck. The databases Medline, Embase, Web of Science (core collection), Scopus and Cochrane Library were searched from inception to April 2021 for studies assessing P53 status and survival. Qualitative analysis was carried out using the REMARK criteria. A meta-analyses was performed and statistical analysis was carried out to test the stability and reliability of results. Twenty-five studies met the inclusion criteria, of which fifteen provided enough data for quantitative evaluation. TP53 mutation was associated with worse overall survival (HR 1.75 [95% CI 1.45–2.10], p < 0.001), disease-specific survival (HR 4.23 [95% CI 1.19–15.06], p = 0.03), and disease-free survival (HR 1.80 [95% CI 1.28–2.53], p < 0.001). Qualitative assessment identified room for improvement and the pooled analysis of all anatomical subsites leads to heterogeneity that may erode the validity of the observed overall effect and its subsequent extrapolation and application to individual patients. Our systematic review and meta-analysis supports the utility of TP53 mutational as a prognostic factor for survival in head and neck squamous cell carcinoma. A well designed prospective, multi-centre trial is needed to definitively answer this question.

## Introduction

Squamous cell carcinomas of the head and neck (HNSCC) are the most common cancers of the head and neck region constituting the 6th most common cancer worldwide (~ 1 million cases per annum^1^). The overarching disease of HNSCC involves tumour arising from the various subsites of the head and neck including, oral cavity, oropharynx, nasopharynx, hypopharynx, larynx and sinonasal mucosa, each displaying variability in presentation, treatment, and prognosis.

The pathogenesis of this disease is multifactorial. However, the majority are associated with tobacco and alcohol misuse, with a notable synergistic effect. A significant minority arise as a result of oncogenic viral infection, particularly Human papillomavirus (HPV; notably in the oropharynx) or Epstein Barr Virus (EBV; commonly in the nasopharynx). Despite variability between subtypes, the incidence of HNSCC is rising and, as a disease cohort, is expected to increase by 30% by 2030^2^. In particular, oropharyngeal squamous cell carcinoma (OPSCC) has doubled in the UK, USA, and Europe in recent decades^2^.

HNSCC exhibits variable responses to conventional treatment. Clinical response rates correlate with survival and are inversely related to primary tumour size, presence and volume of metastatic disease in the cervical lymph nodes and pathological evidence of tumour spread through the lymph node capsule (extracapsular spread). When analysing survival across all age groups and anatomical sites, the 5-year survival for HNSCC has modestly improved from 55 to 66%^3^. However, a subgroup analysis highlights that survival in some anatomical subsites remains stagnant and that this observed overall survival improvement is partially attributable to the emergence of HPV-positive OPSCC^4^.

Despite presenting with clinico-pathological features suggestive of an aggressive phenotype, survival rates are considerably higher for patients with HPV-positive OPSCC, albeit a significant minority (15–20%) will still succumb to their disease^5^. Current epidemiological data suggest that poor outcome correlates tightly with cigarette smoking, hypothesised to be a cause of, and surrogate for, underlying carcinogen-induced mutational load and genetic instability. However, mainstay cisplatin-based chemoradiotherapy (CRT) results in life-changing long-term swallowing disability: up to 20% of patients undergoing CRT require long-term gastrostomy tube feeding^6^. Surgery followed by adjuvant therapy represents a valid alternative treatment option, but clinical decision tools are needed to achieve a consensus. Consequently, there is an urgent need to identify patients who are destined for poor outcome and those for whom treatment de-intensification, with a view to avoiding long term swallowing difficulty, is an option. In contrast, there is an urgent need for the development of new treatments to enhance survival for HPV-negative HNSCC as survival rates remain at 60%^5^.

Whilst data support the prognostic utility of HPV status, no data currently exist to suggest that treatment decision-making based on HPV status is safe and effective^7^. Moreover, the molecular mechanisms by which HPV may contribute to neoplasia development and progression in OPSCC remain poorly understood, with much of our current understanding inferred from data derived from cervical cancer research. Whether such inference is appropriate and relevant is currently unclear.

The *TP53* tumour suppressor is the most commonly mutated gene in human cancer and the p53 protein it encodes plays critical roles in cell-cycle control and apoptosis in response to DNA damage and other cellular stresses^8^. Loss of p53 function, either through disruptive *TP53* mutation or through abrogation by viral oncoproteins (in the case of HPV + disease), occurs with high frequency in HNSCC^9^. Given the pivotal role that p53 plays in regulating cellular response to therapeutic interventions (such as chemotherapy and radiotherapy), it is enticing to hypothesise that disruption of the gene would dictate prognostic significance. To date however, evidence regarding the role of p53 as a prognostic marker for HNSCC remains controversial^10^.

Structurally, p53 is a complex and multifunctional 393-residue protein. It has 3 domains: an N-terminal subunit composed of a transcription-activation domain and a proline rich domain, a central DNA-binding core domain, and a C-terminal domain involved in modulating binding behaviour of the DNA binding domain. The TP53 gene is located on chromosome 17p13.1 and is composed of 25,772 bases.

The consequences of inconsistencies in analytical approaches to identify p53 alterations and variability of cohorts has led to conflicting outcomes. Moreover, considerable variation has been identified in TP53 mutations, with consequent diverse effects on protein function and thus prognostic significance^11^. Loss of *TP53* function has been shown to negatively affect disease outcome in other solid tumours such as bladder carcinomas, while in breast cancer, for example, *TP53* mutation has been linked to improved prognosis in patients treated with chemotherapy but poor prognosis in those treated with hormone therapy^12^. These various responses to treatment in different cancer types appear to have a sound basis in tumour biology, and it is not unforeseeable that such differences to treatment response and *TP53* mutation status may also occur in different HNSCC subtypes.

Immunohistochemistry (IHC) of p53 has been proposed as a surrogate marker for TP53 mutations in diagnostic workup of a number of cancers. However, interpretability of this technique is complicated by mutation-dependent alterations in protein stability and thus immunoreactivity. In the absence of DNA damage, p53 induces its own proteasomal degradation through transcriptional upregulation of the E3 ubiquitin ligase Mouse double minute 2 (MDM2). Thus, WT p53 is inherently unstable under usual conditions and is undetectable through IHC. In cells harbouring deleterious *TP53* missense mutations, MDM2 is no longer induced, and this negative feedback loop is broken, such that p53 persists and is detectable through IHC^13^. This indirect strategy in identifying *TP53* mutations is not suitable for detecting nonsense mutations, which result in truncated non-immunoreactive protein, or for deletions, both of which will result in the absence of p53 staining and appear indistinguishable from WT. Sequencing overcomes these limitations.

We document the outcomes of a systematic review and meta-analysis of the evidence for the prognostic relevance of p53 mutational status assessed using sequencing approaches.

## Methods

This systematic review complies with PRISMA guidelines^14^ and closely followed the criteria of Cochrane Prognosis Methods Group^15^, Cochrane Handbook for Systematic Reviews of Interventions^16^, and Centre for reviews and Dissemination (CRD)’s guidance for undertaking reviews in healthcare^17^.

### Protocol

In keeping with best practice, the protocol, including a priori methodology, was registered in the PROSPERO international prospective register of systematic reviews (www.crd.york.ac.uk/PROSPERO, registration number CRD42021242118), in order to minimize the risk of bias and improve the transparency, precision, and integrity of this study. The protocol adheres to PRISMA-P guidelines to ensure a rigorous approach.

### Research question

This review aimed to examine the relationship between TP53 mutational status, as determined by genomic sequencing, and survival in squamous cell carcinoma of the head and neck (oral, oropharynx, nasopharynx, sinonasal, hypopharynx, larynx). Effective discrimination of clinical outcomes are hypothesised as being suitable to support the design and development of prospective studies seeking to determine the clinical utility of TP53 mutational status as a prognostic (and possibly predictive) biomarker.

### Information sources and search strategy

The search strategy was developed and conducted by a medical librarian. Prior to conducting the searches, the search terms were peer reviewed by another medical librarian according to PRESS criteria^18^. The databases Medline (via Ovid), Embase (via Ovid), Web of Science (core collection), Scopus and Cochrane Library were searched from inception to April 2021 and limited to English language only, with variants of the following terms, which were in the title and abstract fields, as well as in the subject heading term field when these existed in the database. The Medline search is reproduced below; (see Supplementary Document S1 for the full strategies used in all databases):

- (p53* or tp53* or pp53* or TRP53* or TP53BP1* or 53BP1* or p202*).ti,ab. OR Tumor Suppressor p53-Binding Protein 1/ or Genes, p53/ or Tumor Suppressor Protein p53/
- AND
- ((laryn* or oropharyn* or hypopharyn* or "oral cavit*" or mouth or tongue or tonsil* or neck* or head or "sino-nasal" or sinonasal or sinus* or nasomucosa or nasopharyn* or nasal* or nose* or paranasal* or pharynx* or cheek* or lip* or gingiv* or palat*) adj3 (carcinoma* or neoplasm* or cancer* or metastas* or tumor* or tumour*)).ti,ab. OR Laryngeal Neoplasms/ or exp Pharyngeal Neoplasms/ or "head and neck neoplasms"/ or "squamous cell carcinoma of head and neck"/ or exp Nose Neoplasms/ or mouth neoplasms/ or gingival neoplasms/ or lip neoplasms/ or palatal neoplasms/ or tongue neoplasms/

### Eligibility criteria

We considered all human studies that investigated the impact of p53 mutational status on patient survival in head and neck cancers. Application of DNA sequencing technique(s) was necessary for identification of p53 mutational status. Other forms of p53 status determination, such as immunohistochemistry, were excluded. All head and neck subsites were included in this study. Conference abstracts, review papers, letters to the editor and opinion pieces were excluded. Only articles published in the English language were considered.

### Study selection and data extraction

Titles and abstracts were independently screened by two reviewers (SB, GN) against the agreed inclusion and exclusion criteria. Disagreements between reviewers were resolved by consensus. A data extraction tool was used for further analysis of selected full texts—this was initially performed by one reviewer (SB) and verified by a second (GN). Reasons for exclusion were recorded for any publication at full-text stage. Data extraction items included:

1. Article identifiers (author, year, title).
2. Study characteristics (sample size, design, population, inclusion and exclusion criteria).
3. Sequencing technique.
4. Anatomical subsites (oral, oropharynx, larynx, hypopharynx, sinonasal, nasopharyngeal).
5. Outcome measures (survival).
6. Results and conclusions.

### Evaluation of quality and risk of bias

Each publication was critically appraised for both quality and risk of bias using the Reporting Recommendations for Tumor Marker Prognostic Studies (REMARK) criteria^19^. The REMARK criteria consists of a checklist of 20 items^20^, and each item can be further divided into multiple sub-categories^21^. To ensure a consistent interpretation and application of the REMARK criteria, the authors examined the REMARK sub-criteria in tandem and selected those of highest yield: each RCC prognostic biomarker manuscript was evaluated according to 48 separate sub-criteria for a maximum score of 20 points. A full list of the criteria and point per criteria is listed in Supplementary Table S1.

### Statistical analysis

The restricted maximum-likelihood estimator for Tau^2^ was used to assess between-studies variance of treatment effects. Higgin’s *I*^*2*^ was used to assess the proportion of true variance of a weighted outcome, interpreted according to the Cochrane Collaboration, whereby 0–40% was considered as low heterogeneity, 30–60% as moderate heterogeneity, 50–90% as substantial heterogeneity and > 75% as considerable heterogeneity^16^. A p-value of < 0.10 was accepted as a significant Cochrane Q statistic. The generic inverse variance method was used as part of a random-effects model of hazard ratios, from studies reporting the results of multivariate Cox proportional hazards models, to provide an overall estimate of the influence of *TP53* mutation status on overall survival, disease/progression-free survival, and disease-specific survival. Publication bias was assessed through funnel plots of hazard ratios against standard error, and funnel plot asymmetry was quantitatively assessed using Egger’s test. Statistical analysis was performed used the *meta* package on R version 4.0.0. All scripts for meta-analysis are available upon request from the corresponding author.

## Results

**Figure 1:**
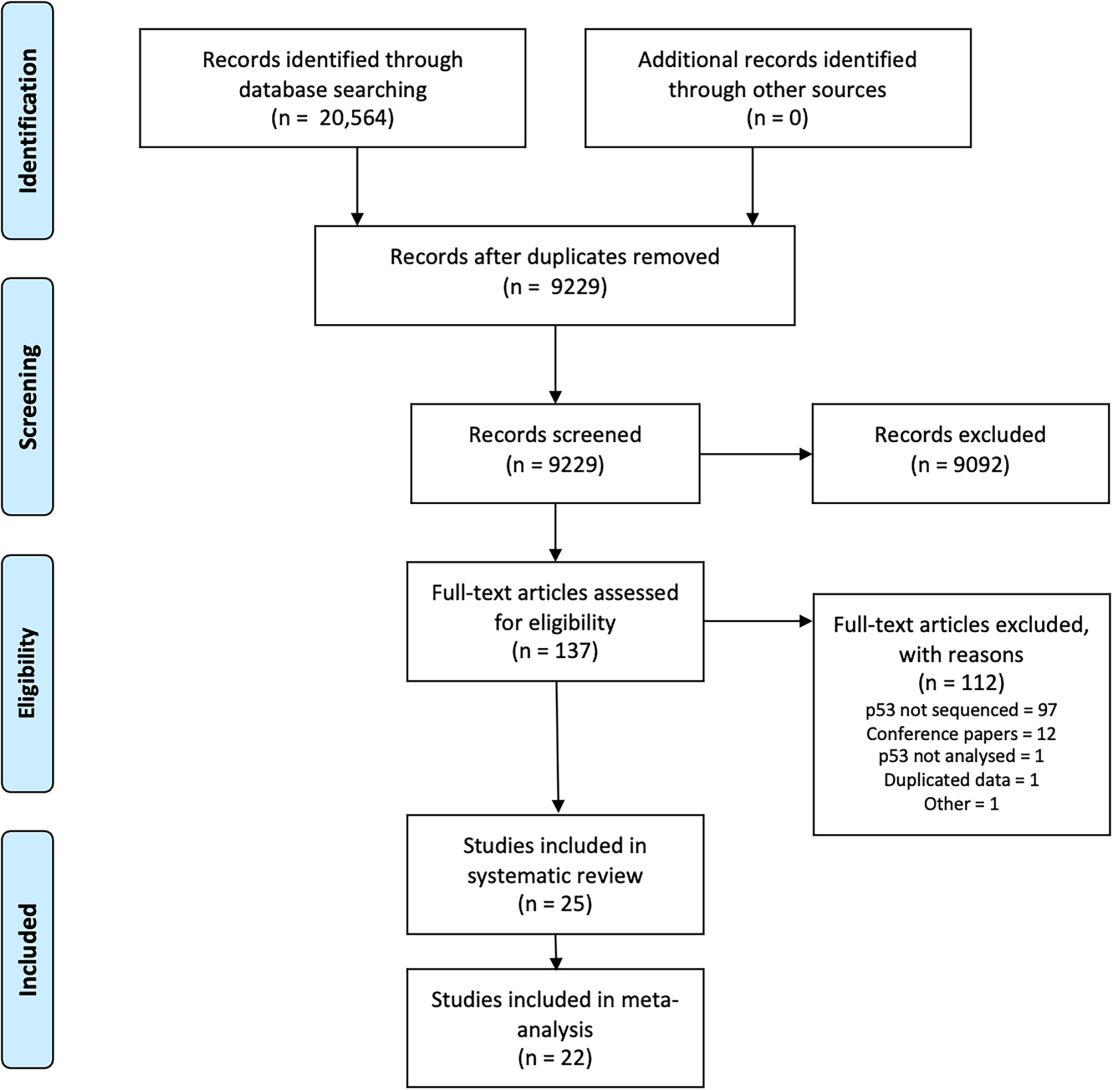
Flow diagram of the process of identification and selection of studies examining the effect of p53 mutation on survival in head and neck cancer.

A total of 9,229 articles were initially retrieved using the search algorithm. After title and abstract screening, 137 records were retained for full-text retrieval, and a total of 25 studies were included at full-text review (Fig. 1) of which 22 provided sufficient data to be included in the quantitative evaluation. 10 of the 25 identified studies were prospective in design, while the remainder were retrospective observational or cohort studies^22–31^.

### Study characteristics

Table 1 summarises the main characteristics of the twenty-five selected studies, published between 1995 and 2020, that carried out p53 sequencing on 3326 tumours with associated clinical outcome data. Sample sizes ranged from 22 to 420 patients. Seventeen studies utilised Sanger sequencing technology to determine p53 status, seven used Next-Generation Sequencing (NGS), and one study used pyrosequencing. Nine studies investigated TP53 mutation in a mixed population of squamous cell carcinoma from all head and neck anatomical subsites. Eight studies restricted investigation of mutations to oral squamous cell carcinoma (OSCC), five studies to laryngeal squamous cell carcinoma (LSCC), and to single studies in oropharyngeal squamous cell carcinoma (OPSCC), nasopharyngeal carcinoma (NPC), and sinonasal squamous cell carcinoma (SNSCC) respectively. Twelve studies were conducted in Europe, six in North America, six in Asia, and one in Oceania. With the exception to Cho et al.^32^ and Poeta et al.^31^, the studies included analysis restricted to primary tumours. Cho et al.^32^ investigated the genomic alterations in 15 nasopharyngeal carcinoma primary tumours as well as the paired primary tumours and recurrent tumours for a further 7 nasopharyngeal carcinoma patients. Poeta et al.^31^ investigated patients with either newly diagnosed or recurrent tumours, with inclusion only if the treatment plan included primary surgical extirpation with curative intent.

**Table 1:** Summarised characteristics of reviewed studies.

Total	25 studies
Year of publication	1995–2020
Total patients (range)	3326 (22–420)
**Sequencing technique**	
Sanger	17
Next-generation	7
Pyrosequencing	1
**Anatomical subsite**	
Mixed	9 studies (1957 patients)
Oral	8 studies (994 patients)
Larynx	5 studies (238 patients)
Oropharynx	1 study (68 patients)
Sinonasal	1 study (57 patients)
Nasopharyngeal	1 study (22 patients)
**Outcome measures**	
Overall survival	21 studies
Disease free survival	14 studies
Disease specific survival	3 studies
**Geographical region**	
Europe	12 studies, 7 countries: Italy, Netherlands, Serbia, Germany, Denmark, Sweden, France
North America	6 studies, 2 countries: USA, Canada
Asia	6 studies, 3 countries: Japan, China, Taiwan
Oceania	1 study, 1 country: Australia

The primary treatment modality was not explicitly reported in 7 of the 25 included studies^23,28,32–36^. Of the remaining studies, twelve investigated patients that underwent surgical resection of the tumour^26,30,31,37–45^. In one study, all patients received primary radiotherapy or chemoradiotherapy without surgery^24^. Five studies included patients who had undergone variable treatments^22,25,27,29,46^.

### Qualitative analysis

**Figure 2:**
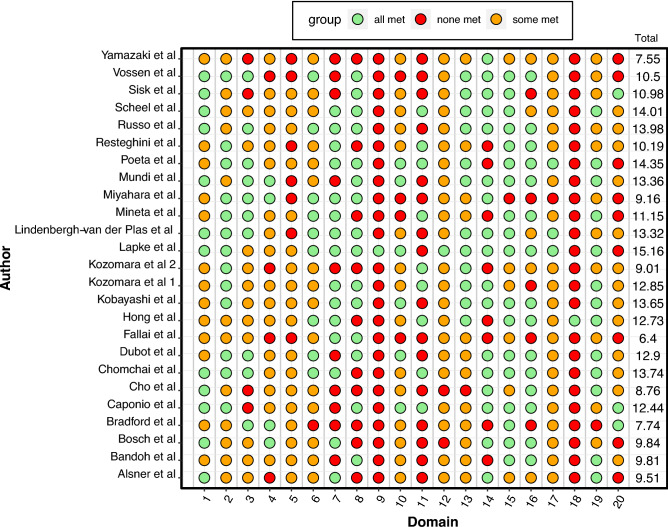
Study fulfilment of the 20-point criteria as set out by the REMARK guidelines^17^. Green indicates the domain was fully met, amber partially met and red none. Total score provided in the rightmost column (maximum score 20).

None of the publications completely fulfilled the criteria set by the REMARK guidelines. The highest scoring paper was awarded 15.16 points out of a maximum of 20 (range 6.4–15.15). Figure 2 presents each study’s fulfilment of the 20-point criterion of the REMARK guidelines with the full score included. Publication bias was assessed visually using funnel plots, identifying no obvious plot asymmetry (Supplementary Fig. S1). This finding was further supported by the results of Egger’s test of plot asymmetry (t(20) = − 1.40, p = 0.18).

### Study outcome measures

Reporting of survival was a necessary requirement for inclusion in this systematic review, however variation in the method/number of measurements was apparent. Most studies (22/25; 88%) reported overall survival^22–33,35,36,38–40,42–46^, 15 studies reported disease/progression-free survival (60%)^24,26,27,29–31,33,34,37,39,41,42,44–46^, and 5 studies reported on disease-specific survival (20%)^22,24,29,36,44^.

Variation in duration of patient follow-up was also variously reported between studies. 8 of the 25 studies did not provide a specific estimate of average follow-up period^23–25,32,33,40,43,45^. Of those studies reporting a median duration, follow-up ranged from 29 months^38^ to 98.4 months^34^.

### HPV status

There was inconsistency in approaches made towards inclusion and reporting of tumour HPV status. Nine studies included HPV-positive tumours in their analysis^22,24,26,33–35,38,42–44,46^. Two studies fully excluded HPV-positive tumours^37,45^, and one paper only specifically investigated HPV-positive cancers^41^. The remaining papers made no mention of HPV status in their inclusion criteria or methodology^23,25,27–32,36,39,40^.

### Outcome

Table 2 summarises the principal findings of each study included in the systematic review. 8 studies reported an analysis of clinical outcomes according to location or category of TP53 mutation^24,26,28,30,31,33,42,44^. Lapke et al.^44^ defined any non-conservative mutation or mutation introducing a stop codon within the DNA binding-domain (DBD) of p53 as disruptive. These disruptive mutations were further characterised as either (i) truncated mutations associated with loss of p53 tumour suppressor activity; or (ii) DBD missense mutations resulting in a possible deleterious gain-of-function. It was found that carriage of DBD missense mutations were associated with a significant reduction in disease-specific survival and disease-free survival compared to wild type p53 (WT), whereby ‘all other mutations’ showed comparable survival characteristics to WT. Caponio et al.^33^ adopted a computational approach to determining the influence of specific TP53 mutations on survival outcomes, identifying DNA-binding domain mutations as a poor prognostic factor in laryngeal cancers (but not other anatomical sub-sites). Further, it was identified that mutations within the hotspot residues R175, H193 and R213 portended a poor prognosis irrespective of HNSCC subsites. In keeping with the findings of Lapke et al.^44^ both missense mutations and those introducing a stop codon were associated with worse overall survival when compared to WT. Both Poeta et al.^31^ and Fallai et al.^24^ characterised TP53 mutations into either disruptive or silent mutations, with disruptive mutations defined as any stop codons, frameshift mutations, and any mutations inside the L2 or L3 domains of the p53 protein resulting in a change in amino acid charge/polarity. In terms of clinical outcomes, Fallai et al.^24^ found no difference in survival according to type of TP53 mutation, whilst Poeta et al.^31^*,* in the largest cohort identified in this systematic review (n = 420 patients), identified a significant reduction in overall survival amongst patients with disruptive mutations. Lindenbergh-van der Plas et al.^26^ similarly used the classification of Poeta et al.^31^ classifying according to the involvement of the p53 DNA-binding domain, and additionally assessed the role of mutation type, namely truncation or missense. Affirming Poeta’s findings, this study identified significantly worse overall survival in the presence of a disruptive mutation, and found that truncating mutations (but not missense) were associated with poorer outcomes on multivariate analysis. In a modest-sized cohort, restricted to oral SCC patients, Yamazaki et al.^28^ showed that mutations in conserved regions of TP53 or within DNA-binding motifs exhibited poorer survival than cases with other p53 mutations. Similarly, the presence of mutations within DNA-binding regions of p53 were strongly associated with locoregional failure, nodal metastasis and distance metastasis. TP53 mutations of exon 5–8, which encode a region important in stabilisation of the protein tertiary structure and the DNA-binding domain, have also been associated with lower survival rates in laryngeal SCC. Russo et al.^30^ in a cohort of 81 stage III and IV laryngeal SCC, found that mutations in exon 5 were an independent prognostic factor for both disease-free survival and overall survival, and that exon 8 mutations were independently associated with overall survival but importantly not relapse.

**Table 2:** Summary table of key outcomes from each study.

Study	Anatomical subsite of HNSCC	Total sample size	Sample preservation method	Clinical outcome measures (survival)	Summary of key outcomes
Kobayashi et al.^32^	Mixed	284	Formalin-fixed paraffin-embedded	Overall Disease/progression-free	– Significantly worse OS, LFFS and DMFS with adverse p53 functional status – Significantly higher local recurrence with adverse p53 with resection margins > 6 mm
Scheel et al.^22^	Larynx	58	Formalin-fixed paraffin-embedded	Overall Disease-specific	– No significant difference in response to chemotherapy according to TP53 mutation status – Significantly worse DSS in TP53 high-risk mutation group - No significant difference in time to indication for surgery according to TP53 status
Lapke et al.^33^	Oral	333	Formalin-fixed paraffin-embedded	Overall Disease/progression-free Disease-specific	– Significantly worse OS and DSS with TP53 mutant vs WT – No difference in DFS between groups – TP53 DBD missense mutations significantly associated with worse OS, DSS and DFS
Dubot et al.^34^	Mixed	122	Snap frozen in liquid nitrogen	Overall	– Significantly worse OS in TP53 mutant vs WT
Caponio et al.^35^	Mixed	415	Various—bioinformatics study on pre-existing datasets	Overall Disease/progression-free	– Significantly worse OS in TP53 mut vs WT (irrespective of mutation) – Significantly worse OS with mutations in B-strand/bridge of p53 secondary structure – Homozygous mutations in TP53 significantly associated with poorer OS and DFS than heterozygous – Transition in p53 interactome in HNSCC with TP53 mutations
Mundi et al.^36^	Oral	123	Immediate DNA extraction following surgical resection; storage on ice for transport	Overall Disease/progression-free	– Significantly worse OS in TP53 mutant group
Cho et al.^37^	Nasopharynx	22	Formalin-fixed paraffin-embedded	Overall	– Mutations in TP53 significantly enriched in patients with disease relapse. Significantly worse OS in TP53 mutant group
Bosch et al.^23^	Mixed	361	Snap frozen in pre-cooled isopentane/liquid nitrogen immediately after resection	Overall	– OS was not significantly different between TP53 mutation classes, irrespective of TNM stage, TP53 mutation type and tumour location
Sisk et al.^38^	Mixed	32	Snap frozen in liquid nitrogen	Overall	– Significantly worse OS in TP53 mutant group, and possible interaction between TP53 and HPV status
Fallai et al.^24^	Oropharynx	68	Formalin-fixed paraffin-embedded	Overall Disease/progression-free Disease-specific	– No difference in survival between TP53 mutant vs WT group – No difference in OS, DFS and DSS between 'disruptive' TP53 mutation vs WT
Bradford et al.^25^	Larynx	26	Formalin-fixed paraffin-embedded	Overall	– No significant difference between TP53 mutant group vs WT group in OS and response to chemotherapy – A significant association between TP53 mutations and advanced tumour stage was identified
Mineta et al.^39^	Mixed	58	Formalin-fixed paraffin-embedded	Overall Disease-specific	– TP53 mutation significantly associated with worse OS
Kozomara et al.^40^	Oral	42	Fresh tumour sections (n = 38) and paraffin-embedded tissues (n = 12)	Overall	– TP53 mutation significantly associated with worse OS
Miyahara et al.^41^	Larynx	28	Fresh frozen (method not specified)	Overall	– TP53 mutation not significantly associated with worse OS – Worse survival identified in patients with p53 protein over-expression
Chomchai et al.^42^	Larynx	45	Snap frozen in liquid nitrogen	Overall Disease/progression-free	– OS was significantly better in patients with a TP53 mutation compared to WT – Trend towards improved DFS with TP53 mutation – No difference in progression-free survival between groups
Lindenbergh-van der Plas et al.^26^	Mixed	141	Snap frozen in liquid nitrogen	Overall Disease/progression-free	– No significant difference in PFS in TP53 mutants (overall) vs WT – Significantly worse PFS on multivariate analysis with truncating mutations in TP53 compared to WT – Disruptive mutations, hotspot mutations and mutations in DNA-binding domains appeared to have no significant influence on OS or PFS
Bandoh et al.^27^	Sinonasal	57	Formalin-fixed paraffin-embedded	Overall Disease/progression-free	– No significant difference in OS and DFS between TP53 mutant and WT groups on multivariate analysis
Yamazaki et al.^28^	Oral	118	Snap frozen in liquid nitrogen	Overall	– No significant difference in OS between WT and mutant TP53 groups – Significantly worse OS in patients with TP53 mutations in conserved regions and DNA-binding domains vs other mutation sites
Alsner et al.^29^	Mixed	114	Formalin-fixed paraffin-embedded	Overall Disease/progression-free Disease-specific	– Significantly worse loco-regional control in patients undergoing radiotherapy with TP53 mutations compared to WT, but not surgery – Significantly worse OS and DFS in patients with mutant p53 vs WT
Russo et al.^30^	Larynx	81	Fresh frozen (method not specified)	Overall Disease/progression-free	– TP53 mutation significantly associated with both poorer OS and DFS – Exon 5 TP53 mutations significantly associated with poorer OS and DFS, and exon 8 mutations associated with OS
Poeta et al.^31^	Mixed	420	Snap frozen at – 80 °C and paraffin-embedded specimens	Overall Disease/progression-free	– TP53 mutation significantly associated with worse OS – Disruptive TP53 mutations demonstrated significantly worse OS than WT, but non-disruptive TP53 mutations did not – Significantly reduced DFS in TP53 mutant group compared with WT, irrespective of mutation type
Hong et al.^43^	Oral	202	Formalin-fixed paraffin-embedded	Overall Disease/progression-free	– TP53 mutation status not significantly associated with loco-regional control, DFS and OS on multivariate analysis
Vossen et al.^44^	Oral	77	Fresh frozen (method not specified)	Disease/progression-free	– No significant difference in PFS according to TP53 mutation status
Resteghini et al.^45^	Oral	67	Formalin-fixed paraffin-embedded (FFPE)	Disease/progression-free	– No significant difference in DFS according to TP53 functional status, though trend towards improved prognosis with TP53 functioning patients
Kozomara et al.^46^	Oral	32	Fresh frozen (method not specified)	Disease/progression-free	– Significantly worse DFS in patients who were TP53 mutation-positive compared to WT

### TP53 mutation status and survival: meta-analysis

**Figure 3:**
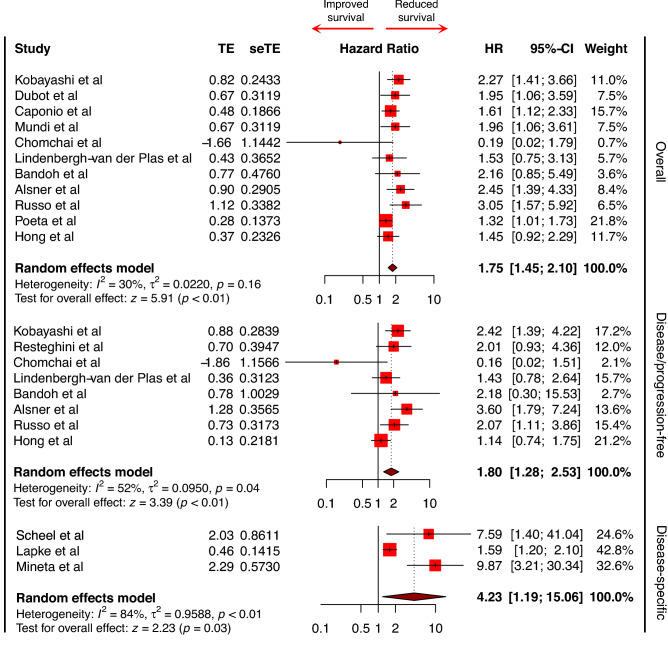
Forest plot graphically representing the meta-analysis on the association between TP53 mutation and survival (overall, disease/progression-free, and disease specific). HR was used as effect size measure. HR > 1 suggests that P53 mutation is associated with reduced survival. Diamonds indicate the pooled HR with corresponding 95% CIs. *HR* hazard ratio, *CI* confidence interval.

15 studies provided data from multivariate cox proportional hazards models amenable to inclusion in a meta-analysis; 11 on overall survival^26,29–31,33,39,42,43,45,46^, 8 on disease-free survival^26,27,29,30,37,39,45,46^ and 3 on disease-specific survival^22,36,44^. See Fig. 3. Random-effects models were used to quantitatively assess overall survival, disease/progression-free survival, and disease specific survival across reporting studies. Between studies, heterogeneity of outcomes was found to be significant for both disease-specific survival (tau^2^ = 0.96; *I*^*2*^ = 83.8% [95% CI 51.3; 94.6%], Q(2) = 12.36, p = 0.002) and disease-free survival (tau^2^ = 0.95; *I*^*2*^ = 51.7% [95% CI 0.0; 78.3%], Q(7) = 14.49, p = 0.04), but not for overall survival (tau^2^ = 0.220; *I*^*2*^ = 30.1% [95% CI 0.0; 65.5%], Q(10) = 14.3, p = 0.16). Random-effects models determined that overall survival (HR 1.75 [95% CI 1.45–2.10], p < 0.001), disease-specific survival (HR 4.23 [95% CI 1.19–15.06], p = 0.03), and disease-free survival (HR 1.80 [95% CI 1.28–2.53], p < 0.001) were significantly worse in patients with *TP53* mutations compared to WT across reporting studies (Fig. 3).

## Discussion

The prognostic value to alteration in the p53 gene (and its function) has been a topic of significant debate in the Head & Neck literature. Previous contrasting reports of the utility of p53 for survival discrimination may, in part, be due to variability in the analytical techniques applied to identify p53 alterations, including identified inconsistencies.

In this systematic review of the literature and associated meta-analysis, we report a comprehensive assessment of TP53 sequencing-defined mutational status as a prognostic factor for survival in patients with HNSCC. The deliberate restriction of p53 status determination to TP53 sequencing technologies alone, allows a significant improvement in understanding by addressing/countering the negative constraints that heterogeneity of mutational status determination has had on prior analyses.

A meta-analysis of the suitable results collated from 15 of the available 25 publications, demonstrated significantly worse outcome with TP53 mutation irrespective of the outcome measure. Although these results are encouraging and represent a progression in the ongoing debate, the authors acknowledge that broadly assigning a global deleterious contribution of TP53 mutation to poor outcomes irrespective of HNSCC subsite or mutation category would constitute a gross oversimplification of the clinical conundrum. Moreover, the problem of heterogeneity persists. Our qualitative analysis highlighted multiple domains of the REMARK criteria which were not sufficiently met. It was particularly notable that guidelines number 9 and 18 (relating to study design and result validation) were poorly met. The number of retrospective studies may, in part, explain this finding as many of the highest scoring papers were those with a prospective study design^22,30,31^.

A further tumour subsite analysis was not possible as many of the papers reporting data from mixed tumours sites failed to provide a clear breakdown based on anatomical location. Without such evidence based on each subsite, it will be difficult to translate any findings into clinical practice as the contribution of any particular head and neck subsite to the observed overall effect is unclear. Furthermore, the majority of the evidence grouped tumours of all stages, critically increasing heterogeneity, as the two most important prognostic factors in HNSCC are primary tumour size (T stage) and regional nodal status (N stage)^47^. It is also important to note that the included papers used a variety of treatment modalities which may influence outcome. Likewise a subgroup analysis of treatment modalities was not possible with the data available. A better appreciation for how *TP53* mutation status may influence treatment response could open the potential for gene status to be used as a predictive biomarker should an interaction exist between *TP53* mutation status and treatment response. Factors such as these should be taken into consideration in the planning of future studies seeking to address these questions.

A failure to address the HPV status of tumours included in various publications similarly contributes to a restriction in the applicability of prognostic determination to specific subsites^48^.

There is now increasing evidence to suggest that differential mutational profiles of the TP53 gene can influence prognosis in several types of tumours^49–51^. Although Neskey et al.^52^ was not included in this systematic review, the results are worthy of mention. This study used the TP53 evolutionary action score to determine high-risk and low-risk mutation. The results demonstrated prognostic significance with high-risk mutations conferring reduced overall and disease-free survival when compared to both low-risk mutations and WT TP53. Amongst studies that met the inclusion criteria, only 8 of the 25 studies in this review reported an analysis of clinical outcomes according to location or type of TP53 mutation^24,26,28,30,31,33,42,44^. Meta-analysis was thus conducted on WT vs mutated TP53, irrespective of mutation location and type.

Circulating tumour DNA (ctDNA) and cell-free DNA (cfDNA) isolated from blood may provide potential for use as blood-based biomarkers in screening, prognostication and monitoring treatment response in HNSCC. However, whilst circulating free DNA (cfDNA) represents an attractive route to explore in the field of precision and personalised oncology, there are current limitations pertaining to testing TP53 mutations in blood. The principal TP53-specific challenge is clonal haematopoiesis which may complicate the interpretation of circulating tumour DNA assays. Clonal haematopoiesis, the accumulation of somatic mutations in haematopoietic stem cells prior to clonal expansion, may result in detectable non-tumour derived mutations in the *TP53* gene, with the potential to reduce the sensitivity and specificity to detect true tumour-derived cfDNA.

Despite the limitations highlighted, our meta-analysis demonstrated that TP53 mutations significantly worsen survival in HNSCC. Whilst these data are compelling, to address the highlighted restrictions in application to clinical practice, a prospective study of TP53 status by sequencing is warranted.

The ECOG-ACRIN 3132 trial (ClinicalTrials.gov Identifier: NCT02734537) offers progress towards this ambition with the incorporation of *TP53* testing as patient stratification. This phase II trial aims to evaluate disease-free survival of patients with locally advanced HNSCC managed with primary surgical resection and adjuvant radiotherapy with or without cisplatin, with consideration to the influence of disruptive *TP53* mutations.

The impact of TP53 mutational state on patient outcomes, stratified by critical clinical and treatment-related variables, remains of significant importance and would require a well characterised, dedicated prospective cohort to resolve understanding. Such an approach would facilitate robust characterisation of the relationship between TP53 status and outcome, in an unbiased manner, critically addressing any potential impact of disease site, stage and/or HPV status, enabling a translation towards clinical practice.

## Conclusion

This review epitomises the difficulties encountered when attempting to determine the impact of TP53 mutation on outcomes in HNSCC based on retrospective data. Our qualitative assessment identified room for improvement for future studies and supports the call for high-quality prospective work to investigate our hypothesis. The pooled analysis of all anatomical subsites leads to a heterogeneity that may erode the validity of the observed overall effect and its subsequent extrapolation and application to individual patients. Furthermore, the inter-study variability with regards to HPV status creates a similar issue. Whilst this review and meta-analysis further supports the hypothesis that TP53 mutational status is of prognostic value in HNSCC, a well-designed prospective, multi-centre trial is needed to definitively answer this question prior to clinical translation.

## Supplementary Information

Supplementary Information 1.

Supplementary Figure S1.

Supplementary Table S1.

Supplementary Information 2.

## Data Availability

All scripts used for data analyses are available upon request from the corresponding author.
