# Supplementary material for: Value of p53 sequencing in the prognostication of head and neck cancer: a systematic review and meta-analysis
Source: Sci Rep. 2022 Dec 1;12:20776. doi: 10.1038/s41598-022-25291-2 (PMC9715723; doi:10.1038/s41598-022-25291-2)
Supplement: Supplementary file 1 — Supplementary Information 1. [file 41598_2022_25291_MOESM1_ESM.docx]

**Supplementary Document S1: Full search strategies and database information**

**Ovid MEDLINE(R) and Epub Ahead of Print, In-Process, In-Data-Review & Other Non-Indexed Citations, Daily and Versions(R) 1946 to April 09, 2021. Searched 12 April 2021**

1. (p53* or tp53* or pp53* or TRP53* or TP53BP1* or 53BP1* or p202*).ti,ab.
2. Tumor Suppressor p53-Binding Protein 1/ or Genes, p53/ or Tumor Suppressor Protein p53/
3. 1 or 2
4. ((laryn* or oropharyn* or hypopharyn* or "oral cavit*" or mouth or tongue or tonsil* or neck* or head or "sino-nasal" or sinonasal or sinus* or nasomucosa or nasopharyn* or nasal* or nose* or paranasal* or pharynx* or cheek* or lip* or gingiv* or palat*) adj3 (carcinoma* or neoplasm* or cancer* or metastas* or tumor* or tumour*)).ti,ab.
5. Laryngeal Neoplasms/ or exp Pharyngeal Neoplasms/ or "head and neck neoplasms"/ or "squamous cell carcinoma of head and neck"/ or exp Nose Neoplasms/ or mouth neoplasms/ or gingival neoplasms/ or lip neoplasms/ or palatal neoplasms/ or tongue neoplasms/
6. 4 or 5
7. 3 and 6
8. limit 7 to english language

**Embase via OVID 1974 to 2021 April 09. Searched 12^th^ April 2021**

1. (p53* or tp53* or pp53* or TRP53* or TP53BP1* or 53BP1* or p202*).ti,ab.
2. *protein p53/
3. 1 or 2
4. ((laryn* or oropharyn* or hypopharyn* or "oral cavit*" or mouth or tongue or tonsil* or neck* or head or "sino-nasal" or sinonasal or sinus* or nasomucosa or nasopharyn* or nasal* or nose* or paranasal* or pharynx* or cheek* or lip* or gingiv* or palat*) adj3 (carcinoma* or neoplasm* or cancer* or metastas* or tumor* or tumour*)).ti,ab.
5. exp *larynx cancer/ or exp *oropharynx cancer/ or exp *hypopharynx cancer/ or *mouth cancer/ or *mouth carcinoma/ or *mouth squamous cell carcinoma/ or exp *pharynx carcinoma/ or *tongue carcinoma/ or *tonsil carcinoma/ or exp *neck cancer/ or *"head and neck cancer"/ or *"head and neck squamous cell carcinoma"/ or exp *paranasal sinus cancer/ or exp *nasopharynx cancer/
6. 4 or 5
7. 3 and 6
8. limit 7 to english language

**Cochrane searched 12 April 2021**

1. (p53* or tp53* or pp53* or TRP53* or TP53BP1* or 53BP1* or p202*):ti,ab,kw (Word variations have been searched)
2. MeSH descriptor: [Tumor Suppressor p53-Binding Protein 1] this term only
3. MeSH descriptor: [Genes, p53] this term only
4. MeSH descriptor: [Tumor Suppressor Protein p53] this term only
5. #1 or #2 or #3 or #4
6. ((laryn* or oropharyn* or hypopharyn* or "oral cavit*" or mouth or tongue or tonsil* or neck* or head or "sino-nasal" or sinonasal or sinus* or nasomucosa or nasopharyn* or nasal* or nose* or paranasal* or pharynx* or cheek* or lip* or gingiv* or palat*) NEAR/3 (carcinoma* or neoplasm* or cancer* or metastas* or tumor* or tumour*)):ti,ab,kw (Word variations have been searched)
7. MeSH descriptor: [Laryngeal Neoplasms] this term only
8. MeSH descriptor: [Pharyngeal Neoplasms] explode all trees
9. MeSH descriptor: [Head and Neck Neoplasms] this term only
10. MeSH descriptor: [Squamous Cell Carcinoma of Head and Neck] this term only
11. MeSH descriptor: [Nose Neoplasms] explode all trees
12. MeSH descriptor: [Mouth Neoplasms] this term only
13. MeSH descriptor: [Gingival Neoplasms] this term only
14. MeSH descriptor: [Lip Neoplasms] this term only
15. MeSH descriptor: [Palatal Neoplasms] this term only
16. MeSH descriptor: [Tongue Neoplasms] this term only
17. #6 or #7 or #8 or #9 or #10 or #11 or #12 or #13 or #14 or #15 or #16
18. #5 and #17

**Scopus searched 12 April 2021**

1. TITLE-ABS-KEY (p53* or tp53* or pp53* or TRP53* or TP53BP1* or 53BP1* or p202*)
2. TITLE-ABS-KEY ((laryn* or oropharyn* or hypopharyn* or "oral cavit*" or mouth or tongue or tonsil* or neck* or head or "sino-nasal" or sinonasal or sinus* or nasomucosa or nasopharyn* or nasal* or nose* or paranasal* or pharynx* or cheek* or lip* or gingiv* or palat*) W/3 (carcinoma* or neoplasm* or cancer* or metastas* or tumor* or tumour*))
3. 1 AND 2

Results limited to English language

**Web of Science Core collection searched 12 April 2021** (Science Citation Index Expanded, Social Sciences Citation Index, Arts & Humanities Index, Conference Proceedings Citation Index – Science, Conference Proceedings Citation index – Social Science & Humanities, Book Citation Index – Science, Book Citation Index – Social Sciences & Humanities, Emerging Sources Citation Index, Current Chemical Reactions, Index Chemicus)

1. TOPIC: (p53* or tp53* or pp53* or TRP53* or TP53BP1* or 53BP1* or p202*)
2. TOPIC: ((laryn* or oropharyn* or hypopharyn* or "oral cavit*" or mouth or tongue or tonsil* or neck* or head or "sino-nasal" or sinonasal or sinus* or nasomucosa or nasopharyn* or nasal* or nose* or paranasal* or pharynx* or cheek* or lip* or gingiv* or palat*) NEAR/3 (carcinoma* or neoplasm* or cancer* or metastas* or tumor* or tumour*))
3. 1 AND 2

Results limited to English language
