# Supplementary figures and images for: Value of p53 sequencing in the prognostication of head and neck cancer: a systematic review and meta-analysis
Source: Sci Rep. 2022 Dec 1;12:20776. doi: 10.1038/s41598-022-25291-2 (PMC9715723; doi:10.1038/s41598-022-25291-2)

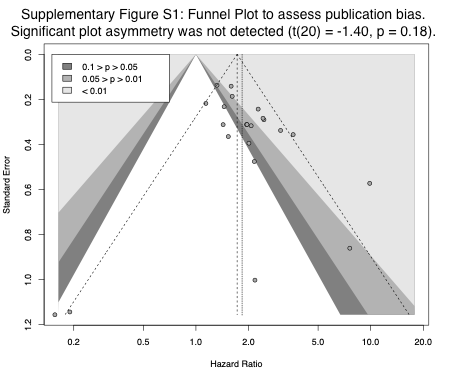

Supplement: Supplementary file 2 — Supplementary Figure S1. [file 41598_2022_25291_MOESM2_ESM.png]
